# Mechanisms of action for stigma reduction among primary care providers following social contact with service users and aspirational figures in Nepal: an explanatory qualitative design

**DOI:** 10.1186/s13033-022-00546-7

**Published:** 2022-08-11

**Authors:** Bonnie N. Kaiser, Dristy Gurung, Sauharda Rai, Anvita Bhardwaj, Manoj Dhakal, Cori L. Cafaro, Kathleen J. Sikkema, Crick Lund, Vikram Patel, Mark J. D. Jordans, Nagendra P. Luitel, Brandon A. Kohrt

**Affiliations:** 1 University of California San Diego, La Jolla, CA USA; 2 Duke Global Health Institute, Durham, NC USA; 3 Transcultural Psychosocial Organization Nepal, Kathmandu, Nepal; 4 King’s College London, London, UK; 5 University of Washington, Seattle, WA USA; 6 Johns Hopkins Bloomberg School of Public Health, Baltimore, MD USA; 7 Patan Academy of Health Sciences, School of Public Health, Kathmandu, Nepal; 8 DePaul University, Chicago, IL USA; 9 Columbia University, New York, NY USA; 10 University of Cape Town, Cape Town, South Africa; 11 Harvard Medical School, Cambridge MA, USA; 12 Harvard T. H. Chan School of Public Health, Cambridge, MA USA; 13 George Washington University, Washington, DC USA

**Keywords:** Mental health, Task-shifting, Primary care providers, Patient involvement, Social contact interventions

## Abstract

**Background:**

There are increasing initiatives to reduce mental illness stigma among primary care providers (PCPs) being trained in mental health services. However, there is a gap in understanding how stigma reduction initiatives for PCPs produce changes in attitudes and clinical practices. We conducted a pilot randomized controlled trial of a stigma reduction intervention in Nepal: REducing Stigma among HealthcAre Providers (RESHAPE). In a previous analysis of this pilot, we described differences in stigmatizing attitudes and clinical behaviors between PCPs receiving a standard mental health training (mental health Gap Action Program, mhGAP) vs. those receiving an mhGAP plus RESHAPE training. The goal of this analysis is to use qualitative interview data to explain the quantitative differences in stigma outcomes identified between the trial arms.

**Methods:**

PCPs were randomized to either standard mental health training using mhGAP led by mental health specialists or the experimental condition (RESHAPE) in which service users living with mental illness shared photographic recovery narratives and participated in facilitated social contact. Qualitative interviews were conducted with PCPs five months post-training (n = 8, standard mhGAP training; n = 20, RESHAPE). Stigmatizing attitudes and clinical practices before and after training were qualitatively explored to identify mechanisms of change.

**Results:**

PCPs in both training arms described changes in knowledge, skills, and confidence in providing mental healthcare. PCPs in both arms described a positive feedback loop, in which discussing mental health with patients encouraged more patients to seek treatment and open up about their illness, which demonstrated for PCPs that mental illness can be treated and boosted their clinical confidence. Importantly, PCPs in the RESHAPE arm were more likely to describe a willingness to treat mental health patients and attributed this in part to social contact with service users during the training.

**Conclusions:**

Our qualitative research identified testable mechanisms of action for stigma reduction and improving clinical behavior: specifically, recovery stories from service users and social engagement led to greater willingness to engage with patients about mental illness, triggering a feedback loop of more positive experiences with patients who benefit from mental healthcare, which further reinforces willingness to deliver mental healthcare.

*Trial registration* ClinicalTrials.gov identifier, NCT02793271

**Supplementary Information:**

The online version contains supplementary material available at 10.1186/s13033-022-00546-7.

## Introduction

Globally, there has been a remarkable increase in mental health task-shifting efforts, with a focus on training primary care providers to deliver mental healthcare [27, 28, 45, 53]. One of the key challenges facing such programs is addressing stigma, which Stuart [56] describes as the greatest barrier to effective mental health programs globally. Stigma is associated with reduced care-seeking by people with mental illness [11, 15], and it contributes to healthcare providers not delivering mental healthcare or providing low quality care for both physical and mental illnesses [1, 6, 22, 42, 43, 44, 62].

Negative attitudes about people with mental illness are widespread among care providers in low- and middle-income countries (LMICs), including perceptions that they are violent, morally to blame, can only be treated by specialists, and that providing treatment puts providers at risk of developing mental illness [22, 36, 37, 47, 52]. In a survey of over 1000 clinical teaching faculty mostly in LMICs, 84% believed psychiatry patients could only be treated in specialized facilities, and 73% reported that psychiatric patients are emotionally draining [55].

Despite evidence regarding high levels of stigma, there is a lack of evidence regarding what works for reducing mental health stigma in LMICs [20, 21, 52]. Although we cannot assume that what works in high-income countries (HICs) is transferable to LMICs [56], evidence from HICs provides some direction regarding what to test in LMICs. For example, there is evidence from HICs that social contact with service users reduces stigma [16, 22, 58], but there is insufficient evidence for this approach in LMICs [21, 52]. Social contact consists of facilitated interaction with mental health service users, such as through sharing recovery narratives [18, 31, 50].

### Theoretical foundations of stigma-reduction interventions

There is a need for multifaceted stigma-reduction interventions that collectively address the three key social psychological aspects of stigma: knowledge, attitudes, and behavior, with the goal of decreasing ignorance, prejudice, and discrimination [12, 22, 57]. Although many studies have reported changes to knowledge and attitudes, behavior has proven most difficult to change [20, 22]. In studies that have assessed behavioral *intentions*, small changes have been documented [14, 56].

Stigma-reduction interventions typically fall into the categories of education, social contact, or protest/advocacy, with education being the most common type [13, 20, 46, 56]. Contact interventions, based on contact hypothesis developed by Allport [3] and others, are increasingly recognized as particularly effective for mental health stigma reduction when facilitated contact occurs between stigmatizing groups and persons living with mental illness [14]. For example, there is evidence that short-term face-to-face contact improves knowledge and attitudes, although data are lacking regarding subsequent behavior change [20].

There is a need for greater attention to mechanisms of action in stigma-reduction interventions [20, 56]. Among existing interventions, only a limited number are strongly theoretically informed [20]. Those that are target important drivers of stigma, including perceived threat or peril stigma, low empathy, and lack of self-efficacy for providing care [2, 19, 48]. For example, one meta-analysis found that contact interventions reduce prejudice primarily through reducing anxiety (e.g., from peril stigma) and increasing empathy, more so than through increasing knowledge [48].

### Theory and concepts explored in in this study

This study reports on an intervention piloted in Nepal entitled REducing Stigma among HealthcAre Providers (RESHAPE) [33, 34]. The intervention draws on theoretical foundations from medical anthropology, social psychology, and social neuroscience [35]. From medical anthropology, we use the “what-matters-most” approach that highlights how valued aspects of one’s culture (e.g., roles, behavioral traits, accomplishments) dictate what types of behaviors and characteristics are stigmatized [29, 64]. From social psychology, we draw on social contact theory, which suggests that intergroup contact reduces stigma and prejudice [48]. From social neuroscience, we draw upon findings that reducing sense of threat and promoting empathy exchange is correlated with lower stigma and prejudice [4]. In addition, from an intervention components perspective, we build upon the finding that stigma reduction for healthcare providers is best accomplished by replacing “myths” with accurate information, reducing stigmatizing attitudes, providing skills to improve clinical competency, increasing willingness to provide care, and ultimately improving quality of care and patient outcomes. For the component focused on reducing stigmatizing attitudes, RESHAPE incorporated Knaak et al.’s [30] “key ingredients” of stigma reduction interventions: personal testimony regarding lived experience, multiple forms of social contact, teaching specific skills, myth-busting, modeling a person-centered approach, and emphasizing recovery.

A key component of the intervention described in this paper is social contact with mental health service users in recovery and their caregivers, through sharing of recovery narratives (i.e., personal testimony) and facilitated discussion. Intergroup (in/out-group) contact theory proposes that positive outcomes are most likely when groups are conferred equal status during the interaction; the interaction involves active co-operation, a mutual goal, a guiding structure, and the opportunity to get to know the out-group member; and it disconfirms negative stereotypes [3, 20]. Many of these elements of positive social contact are difficult to achieve through typical clinical interactions between healthcare providers and service users because of the power differentials and lack of collaboration working toward a mutually valued goal [22]. Additionally, when medical trainees do encounter people with mental illness, it is often those patients who are most severely ill such as in-patients, rather than encountering patients in recovery, which leads to a particular stereotype of the experience of mental illness [7, 20, 22].

An additional component of the intervention described here is the inclusion of aspirational figures, or healthcare providers who have successfully delivered mental healthcare. Gronholm and colleagues [20] argue that such approaches using healthcare providers as stigma change-agents have yet to be fully employed. Such programs run the risk of perpetuating stereotypes, for example if providers focus on stories of encountering violent patients [19, 20, 48]. It is therefore vital that providers portray people with mental illness positively and using a recovery approach [20, 30].

### RESHAPE vs. training-as-usual: quantitative findings

In a pilot cluster randomized controlled trial in Nepal, RESHAPE was compared to a standard mental health Gap Action Program (mhGAP) training (training-as-usual, TAU; see Methods for a description of both training arms). Quantitative findings with 88 prescribers revealed important differences between trial arms [33]. We found that among prescribers, RESHAPE trainees experienced a greater decrease in stigmatizing attitudes, assessed using the Social Distance Scale (SDS, described in detail in Methods; [8]. Mean SDS score changes from pre-training to 16-months were − 10.6 (95% CI − 14.5, − 6.7) in the RESHAPE arm and − 2.8 points (− 8.3, 2.7) in the TAU arm. Additionally, role play-based diagnoses were 78.1% accurate for RESHAPE-trained providers and 66.7% accurate for TAU providers. Real patient diagnoses were 72.5% accurate for RESHAPE, compared to 34.5% for TAU. Finally, RESHAPE providers treated 38% more patients in the 1.5–2 years following the training. Although effect sizes cannot be established given the pilot nature of this study, the findings suggest that RESHAPE’s addition to mhGAP may not only reduce stigma above training-as-usual, but it may also increase treatment provision and improve quality of services in the form of more accurate psychiatric diagnosis.

### Aims

This study draws on qualitative interviews with RESHAPE and TAU trainees to identify potential mechanisms explaining the quantitative differences in stigmatizing attitudes and quality of care found in the pilot cluster randomized controlled trial. We explore trainees’ experiences of the mental healthcare trainings, with a focus on knowledge, attitudes, competence, willingness to provide treatment, and reported behaviors.

## Methods

### Setting

This study was conducted in Nepal, a country with a population of 29 million and a life expectancy of 70 years [10, 63]. Studies have estimated the burden of mental disorders in Nepal to be as high as 22.7% for anxiety and 11.7% for depression [51]. There have been some efforts by both government and non-government agencies to integrate mental healthcare into general health services. However, barriers like lack of proper medications, skilled manpower, and infrastructure, as well as stigma towards mental illness have raised challenges in implementation [5, 41]. As in other LMICs, a significant portion of the population living with mental illness does not receive treatment. Research conducted in Chitwan, a southern region of Nepal—also the site of the current study—found that more than 90% of people with depression and alcohol use disorder did not receive treatment [41]. The total population of Chitwan is 579,984. There is a diverse mix of castes and ethnicities in the area, with several different languages spoken. Chitwan has slightly better health and development indicators than the national average. The under 5 mortality rate for Chitwan is 38.6 per 1000 (national average is 52.9); it also has a higher literacy rate than the national average (79% and 67%, respectively; [10]).

In Nepal, primary healthcare is provided in each district at various levels, with primary care providers staffing health posts, community health units, and urban health clinics. These centers are staffed by different levels of health workers; for simplicity, the cadres of health workers in this study are divided into two groups based on their authority to prescribe medications: prescribers and non-prescribers. Prescribers include health assistants, auxiliary health workers, and medical officers. Non-prescribers include staff nurses and auxiliary nurse midwives [40]. Both prescribers and non-prescribers participated in the pilot cluster RCT. Data on the proof-of-concept phase have been published for both prescribers and non-prescribers [35]. However, only the prescriber quantitative data in the randomized controlled phase have been published to date [33]. The current qualitative analysis includes both prescribers and non-prescribers.

In the study site of Chitwan, there are 41 health facilities (2 hospitals, 3 primary healthcare centers, and 36 health posts/sub-health posts). Since 2011, the district has been a site of the multi-country Programme to Improve MEntal Healthcare (PRIME) [23, 42]. PRIME integrates mental healthcare into primary healthcare by training both prescribers and non-prescribers. The local partner organization Transcultural Psychosocial Organization (TPO) Nepal has been conducting mental health research, interventions, advocacy, and service-provision for more than 15 years in Nepal [60]. TPO Nepal partners with the government in similar programs aimed at integrating mental healthcare into primary and community healthcare [24, 25, 39].

### Training

Participants in this study were primary care providers who participated in either a training-as-usual (TAU) arm or the modified RESHAPE training arm. All received 9 days of mhGAP training (prescribers) or 5 days of psychosocial training (non-prescribers; see Table 1). Trainings covered psychosocial skills and, for prescribers, additional modules for diagnosis and treatment of four mhGAP disorders: psychosis, epilepsy, depression, and alcohol use disorder, as well as the suicide prevention module. The RESHAPE training followed the same model as the TAU arm but with trained service users, their caregivers, and aspirational figures serving as co-facilitators on different days of the training [26, 35, 49]. The service users’ mental health conditions were matched with the mental health condition of focus for the day, and they were involved in two major activities: sharing recovery narratives and formal/informal interaction with health care workers. During days of their involvement, the service users participated in about 1 h of presentation and Q&A, and they attended the full day so they also participated in social activities and energizers and meals. Aspirational figures are PCPs who have previously been trained on mental healthcare and have shown a commitment to delivering the services after training, i.e., they are role models that other PCPs should aspire to emulate. Aspirational figures received approximately 4 training sessions on sharing recovery stories from their perspective and on conducting myth-busting [35].

**Table 1:** Contents of Training as Usual (TAU) vs. RESHAPE modified training curriculum

Training day	Primary care non-prescribing staff		Primary care prescribing staff	
	Training as Usual (TAU) 5-Day Curriculum	RESHAPE modifications to training	Training as Usual (TAU) 10-Day Curriculum	RESHAPE modifications to training
1 (6.5 h)	• Introduction to PRIME • Pre-test • Introduction to mental health and psychosocial concepts	None	• Introduction to PRIME • Pre-test • Introduction to mental health and psychosocial concepts	None
2 (6.5 h)	• Introduction to MNS problems, causes, and symptoms • Basic principles of psychosocial support for patients with MNS problems; characteristics of helpers • Psychosocial support skills (emotional support, psychoeducation, case management)	Two non-prescriber aspirational figures present recovery stories and common myths about MNS disorders: • Mental illness cannot be treated.Only some people can get mental illness • Mental illnesses are contagious • Mental illness can only be treated with shots and pills • Giving advice is the same thing as doing psychological counseling • All people with mental illness are violent.If you ask someone about suicide, that increases the risk they will kill themselves • Caring for people with mental illness makes you mentally ill	• Introduction to MNS problems, causes, and symptoms • Basic principles of psychosocial support for patients with MNS problems; characteristics of helpers • Psychosocial support skills (emotional support, psychoeducation, case management)	Two prescriber aspirational figures present recovery stories and common myths about MNS disorders • Mental illness cannot be treated • Only some people can get mental illness • Mental illnesses are contagious • Mental illness can only be treated with shots and pills • Giving advice is the same thing as doing psychological counseling • All people with mental illness are violent • If you ask someone about suicide, that increases the risk they will kill themselves • Caring for people with mental illness makes you mentally ill.
3 (6.5 h)	• Introduction to communication skills • Verbal communication skills (questioning, reflecting feelings, summarizing, paraphrasing) • Role plays of communication skills • Non-verbal communication skills	Two persons with lived experience of MNS disorders present PhotoVoice recovery narrative, participate in question and answer session, and participate in activities throughout the day with primary care trainees Didactic session on stigma and discrimination	• Introduction to communication skills • Verbal communication skills (questioning, reflecting feelings, summarizing, paraphrasing) • Role plays of communication skills	Two persons with lived experience of MNS disorders present PhotoVoice recovery narrative, participate in question and answer session, and participate in activities throughout the day with primary care trainees
4 (6.5 h)	• Role plays of communication skills • Depression: causes, symptoms, and referrals • Psychosis: causes, symptoms, and referrals • Epilepsy: causes, symptoms, and referrals	Two persons with lived experience of MNS participate in communication role plays and activities throughout the day with primary care trainees	• Non-verbal communication skills • Role plays of communication skills • Emotional support steps and role plays • Psychoeducation steps and role play	Two persons with lived experience of MNS participate in communication role plays and activities throughout the day with primary care trainees Didactic session on stigma and discrimination
5 (6.5 h)	• Alcohol use disorder: causes, symptoms, and referrals • Psychoeducation and case management steps and role play • Health management information system • Supervision processPost-test	Collaborative problem-solving session with two persons with lived experience of MNS disorders, two aspirational figures, and trainees to discuss expected challenges and potential solutions	• Case management steps and role playIntroduction to mhGAP • Basic information about MNS disorders included in mhGAP	None
6 (6.5 h)			• Psychiatric history takingEpilepsy assessment, diagnostic criteria, and management • Clinical patient evaluation	One person with lived experience of epilepsy presents PhotoVoice recovery narrative, participates in question and answer session, and participates in other activities throughout the day
7 (6.5 h)			• Depression assessment, diagnostic criteria, and management • Clinical patient evaluation	One person with lived experience of depression presents PhotoVoice recovery narrative, participates in question and answer session, and participates in other activities throughout the day
8 (6.5 h)			• Psychosis assessment, diagnostic criteria, and management • Clinical patient evaluation	One person with lived experience of psychosis presents PhotoVoice recovery narrative, participates in question and answer session, and participates in other activities throughout the day
9 (6.5 h)			• Alcohol use disorder assessment, diagnostic criteria, and management • Clinical patient evaluation	One person with lived experience of alcohol use disorder presents PhotoVoice recovery narrative and participates in question and answer session Collaborative problem-solving session with two persons with lived experience of MNS disorders, two aspirational figures, and trainees to discuss expected challenges and potential solutions
10 (6.5 h)			• Health management information system • Supervision process • Post-test	None

The RESHAPE arm included myth-busting by aspirational figures, which debunked common myths regarding mental illness like “mental illness cannot be treated” and “mental illnesses are contagious” (see Day 2 in Table 1). Some of this myth-busting might have been present implicitly in the original training arm, such as implicitly addressing the myth that mental illness cannot be treated or that it can only be treated with shots and pills. However, the explicit myth-busting discussion only occurred in the modified arm, and it was delivered by an aspirational figure.

### Data collection

Four to five months following the training, a subset of trainees was invited to participate in in-depth interviews. The health workers were selected through a random selection process—where each health facility was assigned one interview from a health worker who had participated in training—followed by a quota sampling process. Within facilities, health workers were selected based on their training and position (MBBS doctor, prescriber, non-prescriber), with effort to maintain gender balance. We oversampled from the RESHAPE arm because the focus of the current study was on the modified training. Some TAU participants had been interviewed previously in formative work at the inception of the PRIME research in Chitwan [9]. At that time in PRIME, there were general interviews with PCPs about their willingness to engage in mental health service provisions and their experiences in initial mental health trainings.

Interview participants included 8 providers from the TAU arm and 20 providers from the RESHAPE arm (7 of whom participated in a pilot of the RESHAPE training). The interview guide was developed and piloted by Nepali and US researchers. Interview topics included perceptions of the training, perceptions of mental illness, and experiences treating mental illness prior to and following the training (see Additional file 1). Interviews asked about mental health generally, though the training focused on depression, psychosis, epilepsy, and alcohol use disorder. For the health workers in the RESHAPE arm, additional questions were asked about their experience with the service users and their caregivers during the training. Interviews were conducted in Nepali by native-speaking researchers, lasted approximately 45 min to an hour, and were audio-recorded.

The study team included researchers from Nepal and the US, with mentorship from researchers from India, South Africa, and the US. Data analysis was conducted by Nepali and US researchers with training in anthropology, global health, public health, and psychology. These analysts were either employed by or partnering with the NGO that delivered the trainings, and the broader research team included researchers and clinicians who designed both the original and modified trainings. Ethical approval for this study was provided by the Nepal Health Research Council, Duke University institutional review board, and George Washington University institutional review board. Participants were informed about the goals and scope of the interview, benefits and harms, and confidentiality, and both verbal and written consent were provided, as well as consent to be audio-recorded.

### Analysis

We used thematic analysis focused on inductive themes. Interviews were transcribed into Nepali by the interviewer and translated into English for analysis. Transcripts were reviewed independently by 3 Nepali and 4 American team members to identify themes, which were then discussed after each transcript and codes agreed upon. A codebook with initial code definitions was developed and iteratively adjusted as additional themes were identified or existing themes were expanded. Review of transcripts continued until 20% had been reviewed, at which point no new themes arose. Three coders completed an inter-coder agreement exercise, applying the codebook independently to an interview, discussing disagreements and coming to consensus on coding, adjusting the codebook as needed, and repeating the exercise until 80% agreement was reached. All remaining interviews were coded by one of the three coders.

For this article, text segments coded for stigma and attitudes/willingness to treat were reviewed and code summaries developed. Findings from trainee interviews were compared systematically to identify differences by gender, role (prescriber/non-prescriber), training arm, or quantitative change in stigmatizing attitudes (see below). Because quantitative data had not been analyzed before code development and coding were undertaken, those findings did not inform our coding process. Rather, quantitative findings were used to inform later stages of analysis, specifically structured comparisons between those with greater or lesser changes in stigmatizing attitudes. In our results, we describe patterned differences in how or how often subgroups described themes of interest. Because subgroup sizes differed, we focused on proportional differences in commonality of themes.

In order to categorize interview participants into subgroups based on changes in stigmatizing attitudes, we drew on quantitative data from the pilot trial. On the first and last days of training, all participants completed a suite of quantitative assessments that included the Social Distance Scale (SDS) [8] adapted for use in Nepal [34]. The SDS included twelve questions answered as willingness to agree, with a Likert scale ranging from 1 (Definitely willing) to 6 (Definitely unwilling). Questions covered themes like interpersonal relationships (e.g., willingness to have people with mental illness as their neighbor, friend, life partner) and interactions (e.g., willingness to provide healthcare and invite them to various personal and community activities). SDS scores were summed, with missing items accounted for through mean imputation at the individual level. Median pre-training scores and median pre/post change scores were calculated for all training participants across both training arms (n = 205), among whom 95 were non-prescribers and 110 were prescribers.

Interview participants’ pre-training SDS scores were then categorized as above or below the median, with higher scores representing greater stigmatizing attitudes as reflected through a desire for greater social distance. Among those with high pre-training scores, their post-training change scores were categorized as above median change (‘Large SDS score decrease’), below median change (‘Small SDS score decrease’), or ‘Increase in SDS score’.

## Results

### Participants

Our sample included roughly 30% of participants from the TAU arm and 70% of participants from the RESHAPE arm, including some who participated in the RESHAPE pilot (Table 2). Consistent with the gender distribution of healthcare roles in Nepal, all of the male participants were prescribers, and there were 6 female prescribers and 9 female non-prescribers.

**Table 2:** Characteristics of primary care providers interviewed (N = 28)

Characteristic	n (%) or Mean (range)
Women Men	16 (57%) 12 (43%)
Age	37 (22–54)
Prescriber Non-Prescriber	18 (64%) 10 (36%)
Years working in health system	14 (2 months – 28 years)
Training arm TAU RESHAPE	8 (29%) 20 (71%)

*TAU* training-as-usual, *RESHAPE* REducing Stigma among HealthcAre Providers

**Fig. 1:**
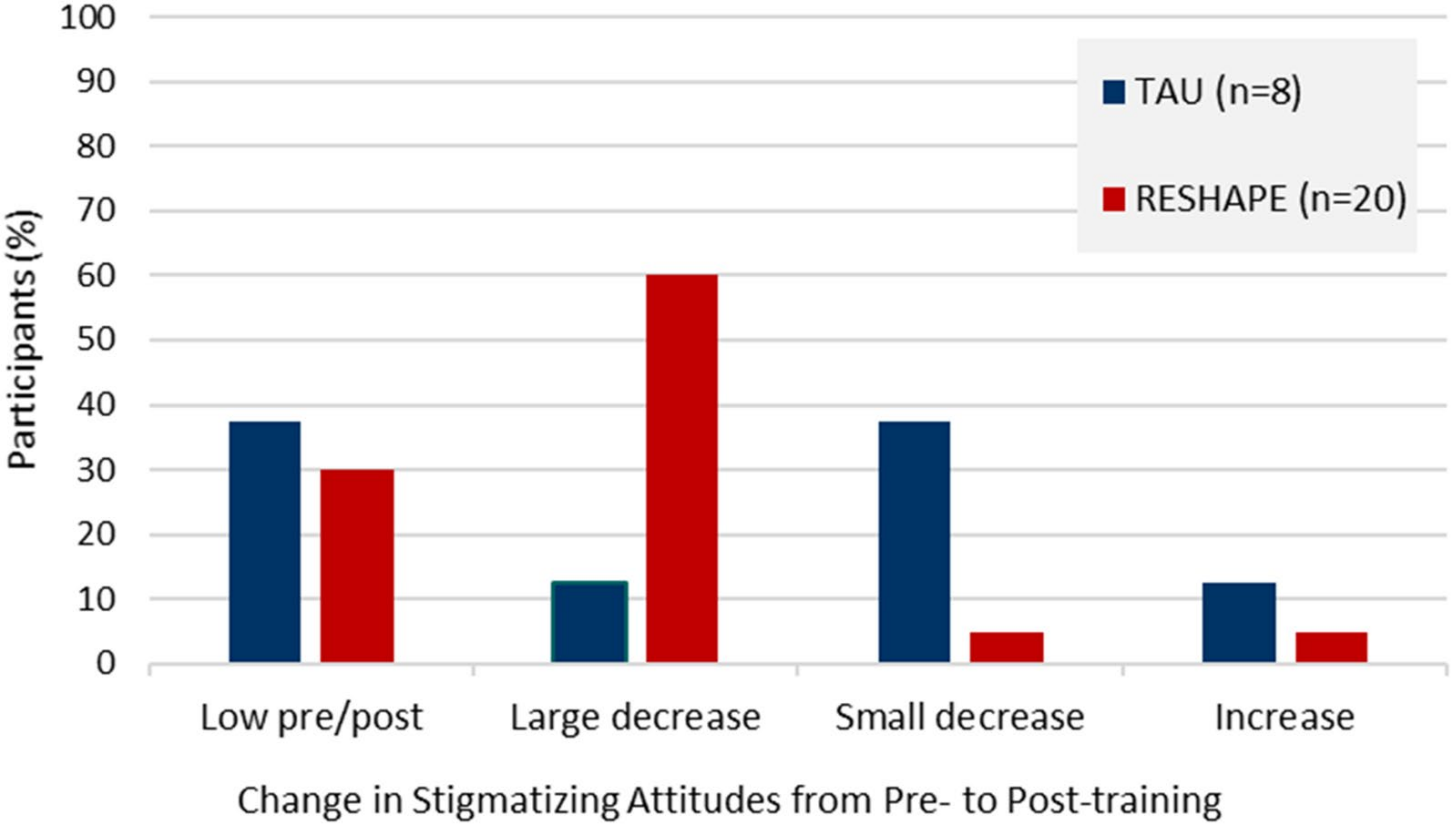
Change in Social Distance Scale (SDS) Scores Pre- and Post-Training by Training Arm (N = 28)

Figure 1 indicates how interview participants were categorized into sub-groups based on changes in stigmatizing attitudes, using pre-post scores on the SDS. Among those with high (above the median) stigmatizing attitudes pre-training, those in the RESHAPE arm almost all had a large decrease in SDS scores following training. There was a more mixed outcome for those in the TAU arm, with most having a small decrease in SDS score.

### Interview findings

#### Societal context of stigma

Much of the discussion of stigma across interviews centered on mental illness stigma and exclusion in society at-large, which providers described as a major barrier to care-seeking and treatment. Providers described that people are laughed at, teased, or cursed by children. They described name-calling and stigmatizing words: “*In the village, one or two people tend to show signs of madness. But we used to call people mad even for the tiniest of things or even a simple different behavior. If the behavior is extreme, then obviously, we dubbed them insane*” (RESHAPE Female prescriber, Large decrease in SDS score^1^). One provider asserted that these attitudes and behaviors exist because society is poor, illiterate, uneducated, and doesn’t empathize.

Several providers described that people cannot talk about mental illness due to this pervasive social stigma. One provider described that families will lock individuals in a room and fear that their daughters will not be able to marry. He also described that “elite” will go to a farther hospital for treatment so that they are not seen seeking mental healthcare by people they know. Providers gave mixed opinions regarding the influence of religious or spiritual perspectives on stigmatizing attitudes. Some described religious or spiritual explanations as contributing to stigma, with attributions of possession or causation by a god or spirit being harmful. For example, a provider explained that epilepsy is considered a curse by God due to sins in one’s past life. In contrast, another provider explained that people will attribute mental illness to magic spells because it is less stigmatizing and that, in contrast to taking mental health medication, going to a traditional healer is seen as being treated by God.

#### Providers’ attitudes and behaviors prior to training

“*When we didn’t have the training—or even when we knew a little after training but there were no medicines—we felt like we are in a condition of soldiers who are going to war without weapons: we are not able to do anything, and we want to defend*.” (RESHAPE Male prescriber, Low pre/post SDS score).

When interviewed post-training, providers described what their own stigmatizing attitudes had been regarding mental illness prior to the training. Most of these attitudes reflected various forms of social distance. For instance, several providers simply stated that they used to call such patients “mad” or “psycho” and refer them, ignore them, or run away from them. Similar statements were made regarding colleagues, with two providers noting that others had either avoided taking detailed histories of mental health patients, resulting in misdiagnoses, or made excuses to avoid seeing the patients at all. One recounted that both she and her colleagues felt that people with mental illness “*can’t be close to us […] Because of their condition, the society says things about them, and they have to live away from the society*” (RESHAPE Female prescriber, Large SDS score decrease).

Several providers described feeling fear towards people with mental illness, particularly fear of being harmed. Female participants were more likely to report having been afraid of patients or avoiding them prior to training. Additionally, trainees in the TAU arm were more likely to describe having thought mental health patients were violent pre-training. In contrast, providers who scored low on the SDS prior to training were less likely to report having been afraid of mental health patients, having seen patients as violent, or having called them names like “mad.”

Trainees described these stigmatizing attitudes as embedded in a context of lack of knowledge, experience, and confidence regarding mental healthcare. Several indicated that they had no idea how to deal with mental health patients, with some indicating a sense that mental illness was not treatable. This pre-training attitude was more commonly described by women, RESHAPE trainees, and those who demonstrated a large decrease in SDS scores. One provider explained, “*Before receiving training, we feared the people who had mental illness. We used to think they might harm us and that we cannot do anything for them. We thought we had to do their treatment by tying them up, locking them in a room*” (RESHAPE Female non-prescriber, Large SDS score decrease). In contrast, one provider described that pre-training, he already knew not to discriminate against any type of patient, and he used to counsel or refer patients. Various sources and forms of misunderstanding were described as contributing to these stigmatizing attitudes. For example, one provider described that in his medical training, epilepsy was explained as hysteria. Another recounted that he used to believe that mental health patients were simply being dramatic and seeking attention.

Several trainees partly attributed their former stigmatizing attitudes to either lack of prior encounters or negative prior encounters with mental health patients. A few providers described themselves as uneasy or “extremely scared” when they first encountered patients with epilepsy or psychosis and linked these feelings to their lack of confidence. Several others described negative experiences, with patients refusing medicine or throwing it back at them, shouting, hitting, or losing their temper: “*While I was doing my medical school (MBBS), there was a case of alcohol use disorder. He slapped our professor very hard. I remember it was a violent case and because he had already gone into alcoholic psychosis, he didn’t know what he was doing*” (TAU Male prescriber, Small SDS score decrease). These examples of negative encounters were more common among providers whose pre-training SDS scores were high but decreased post-training.

A few providers mentioned others’ experiences as influencing their own attitudes. For example, two providers referenced rumors or name-calling directed at mental health providers (e.g., *pagal ko dactar*, “crazy person doctor”), while another recounted having his other patients shout because he spent too much time with one mental health patient.

#### Providers’ attitudes after training

Providers described a range of ways that their attitudes and perceptions changed post-training, with largely consistent findings between TAU and RESHAPE providers. They described learning that anyone can have a mental illness and that mental health patients are “like us.” Providers’ framing of their changed attitudes reflected shifts in social distance. For example, two trainees referred to mental health patients as our brothers and sisters or as part of society, with another adding that just as people should not discriminate based on caste, they should not discriminate against mental health patients. Providers also described coming to learn that patients can be treated easily and come to live well: “*After training, I have been able to understand that they are also like us and they can come to receive treatment in an easy way, they can be well and can live the life they had before*” (RESHAPE Female non-prescriber, Large SDS score decrease). Many of the providers framed this discussion by saying that patients’ lives can go back to “normal.” RESHAPE trainees sometimes provided specific examples of recovery narratives: “*I felt very happy to see the service users. They shared about their situation when they were struggling with mental health problem and their situation now. They shared that before they used to drink alcohol and fall down on the street, but now they have improved; their family condition has improved too*” (RESHAPE Female non-prescriber, Large SDS score decrease).

In describing their changed behaviors, many providers explained learning that one must approach mental health patients with care,^2^ empathy, and respect: “*We used to laugh at them and named them mad, but after the training […] I have learned that we should not make fun of them; we should listen to their feelings. After taking the training, I feel that I can understand his/her feelings and help him/her. I am feeling bad for my behaviors towards them*” (TAU Female non-prescriber, Increase in SDS score). Many providers emphasized the importance of listening, counseling, and understanding and not humiliating or offending patients. This emphasis on listening and counseling was more common among providers who had low SDS scores pre-training or whose scores decreased post-training. Learning to avoid name-calling or speaking carelessly was also emphasized, with providers avoiding terms like “mad” because they now sympathize with patients. Three providers noted that talking to the family or guardian is beneficial, both to help them understand that the patient can get better and to support their care provision.

#### Knowledge, confidence, and willingness to treat

Following the training, most providers expressed willingness or motivation to treat mental illness, with such attitudes expressed more commonly among RESHAPE trainees. Providers from both arms described that, before the training, they were afraid to treat people with mental illness or would refer them. In contrast, after the training, they expressed motivation, confidence, and enthusiasm for treating people with mental disorders: “*We had basic knowledge, but now the training has added more energy to work*” (TAU Female prescriber, Low pre/post SDS score). Several providers mentioned specific individuals in their neighborhood whom they have treated or would like to treat, and one expressed interest in learning to counsel people who have attempted suicide.

Expressions of willingness to treat were more common among trainees from the RESHAPE arm, the majority of whom verbalized a willingness to treat mental health patients. About half of interviewees from the TAU arm expressed willingness to treat. Willingness to treat was also somewhat more common among prescribers compared to non-prescribers.

Several reasons were mentioned as leading to this increased motivation to provide treatment. Some trainees mentioned feeling that they can provide treatment now that they have learned specific skills, such as identification of mental disorders and counseling. Building confidence due to knowledge gained from the training was cited frequently by health workers. They mentioned that the training boosted their confidence to identify patients with mental health problems, even if they came to the health facility with physical complaints: “*Before the training, I couldn’t be this close to the patient. I couldn’t really figure out what the case was before. When there were serious cases that was noticeable then I couldn’t even confront them out of fright. Now, I can tell what person has what symptoms when the person has psychosis or depression or as such*” (TAU Female prescriber, Large SDS score decrease). Providers also described feeling confident about delivering the correct treatment, such as providing medication and/or counseling when needed and referring patients to specialists.

Providers also attributed their increased confidence to learning that treatment is possible, which RESHAPE trainees directly linked to service users’ and aspirational figures’ examples: “*When [service users] participate, we can believe that counseling can help people. This motivates me to do the same when I return to my organization*” (RESHAPE Female non-prescriber, Large SDS score decrease). One provider described that hearing from health workers who have provided mental healthcare motivated them to feel that they can do it too. Compared to the TAU arm, RESHAPE trainees referred more frequently to learning about interacting with patients, how to deal with patients safely, and creating a better environment for patients to be open about their problems, for example, “*The problem is because they can’t open up, so if we can create the environment, then people with mental problems won’t have to hide. So helping them cope with the illness and treating them, it is necessary*” (RESHAPE Female prescriber, Low pre/post SDS score).

Several providers from both training arms framed their discussion of changed attitudes in terms of changed understandings regarding causation of mental illness. Some described coming to understand that mental illness does not “come out of nowhere,” with another adding that it is not a curse or possession. Several providers, most of them men, emphasized the importance of identifying underlying reasons for individuals’ mental health problems: “*Now, when I see someone with these types of problems, I wonder what happened to him and how, the stages of the illness, and that we could have helped them had they came to us earlier. They would get back to their normal lives, the society, and their families*” (RESHAPE Female prescriber, Large SDS score decrease).

Another reason for increased willingness to treat in both training arms was a desire to help others return to normal or avoid advancing to a more severe illness. One provider simply stated that it feels good when patients improve and express gratitude. Another commonality was respondents framing their motivation to treat in the context of their responsibility. Several expressed that as a healthcare provider or simply a fellow human, they have a responsibility to provide care, for example, “*The government has invested so much funding, and it’s not ethical for us not to work*” (RESHAPE Male provider, Increase in SDS score). One provider described helping people return to society as a “righteous” thing to do.

#### Patient encounters

Providers from both training arms described that their changes in attitude were also related to shifts in patients’ behavior, in a positive feedback loop. Several providers described that patients were sharing more openly now and that those who had previously hidden their disease were coming for help. This gave providers confidence, and some reported feeling happy and proud upon seeing a patient benefiting. In the other direction, providers attributed such changes in patients’ openness to the provision of treatment, which led people to see mental illness as treatable like any other problem: “*The ones who are being counseled and take medicines are doing well too. This makes me very happy. I never thought so many [patients] would come to our area. What I found out was if you provide the service, people will come and seek help*” (RESHAPE Female non-prescriber, Small SDS score decrease). One participant provided a specific example of people who saw their neighbor improve and then themselves asked for help. At the same time, two providers described that creating an environment where patients do not feel the need to hide remains an ongoing challenge. One noted that people in villages are skeptical or resistant to seeking mental health treatment, while another added that, although he does not experience stigma, he also has not been appreciated by communities for providing mental healthcare.

#### Providers’ experiences of stigma

When asked about their own experiences of stigma post-training, all providers except one who were asked the question stated that they and their families did not experience stigma. One provider (TAU Female non-prescriber, Low SDS score pre-training) described people saying that mental healthcare providers “belong in the same category” as their patients. In addition, another provider noted that he has not yet gone “to the field,” implying that he might expect to experience stigma in communities. In contrast, one provider anticipated that telling the community about mental health service provision would engender positive responses, and two others described people already viewing providers in a positive way for providing mental health services. One RESHAPE trainee attributed the lack of stigma towards providers to people being educated, while two others explained that being a general practitioner—hence treating a wide range of illnesses—enables them to avoid stigma associated with mental healthcare specialists. While two providers stated that the mental health service does not affect other services, one described not having time for new psychosis patients because of existing patients. See Table 3 for a summary of interview findings.

**Table 3:** Summary of Themes from Interviews with Trained Primary Care Providers at 5-months post-training (N = 28)

	Overall findings	Differences by training arm^1^
Societal context of stigma	• People with MI laughed at, teased in society • People don’t talk about MI because of stigma • Seek care for MI far away where don’t know people	
Reported attitudes and behaviors before training	• Used stigmatizing language (e.g., “mad”) • Avoided, feared MH patients • Lacked knowledge, experience, confidence with MH • Lack of or negative prior encounters with MH patients	• TAU providers more often reported having considered MH patients violent • RESHAPE providers more often reported thinking MI was not treatable
Attitude changes after training	• Anyone can have MI; they are “like us” • MI is treatable; patients can return to “normal” • Should treat patients with care, empathy, respect	• As in the overall quantitative sample, RESHAPE interview participants more likely to experience large decrease in stigmatizing attitudes • RESHAPE providers referred to recovery narratives in describing changed understanding
Willingness to treat after training	• Motivation, confidence, enthusiasm to treat MI • Confidence attributed to increased knowledge, skills • Motivation attributed to greater understanding of causation of MI • Responsibility, desire to help return to “normal”	• RESHAPE providers more likely to express willingness to treat • RESHAPE providers more often described specific skills learned (e.g., how to interact, promote safety, and encourage openness)
Patient encounters during and after training	• Treatment provision/success → greater patient openness → greater provider confidence and openness → treatment provision/success (positive feedback loop)	• RESHAPE providers described positive impact of personal testimony from service users, caregivers, and aspirational figures (current MH providers)
Providers’ experience of being stigmatized after training	• All except 1 reported not having experienced stigma • Mixed expectations regarding stigma if they were to advertise MH services	

1. Because of the purposive sub-samples used, some patterned differences were noted between TAU and RESHAPE participants; these differences between training arms are descriptive and not statistically tested or inferential

*MH* mental health,* MI* mental illness,* RESHAPE* REducing Stigma among HealthcAre Providers,* TAU* training-as-usual

## Discussion

To the best of our knowledge, this is one of the first studies to qualitatively explore possible mechanisms of change in a social contact intervention for stigma reduction among primary care providers in an LMIC setting. This explanatory phase of a mixed-methods study of providers in Nepal identified important ways that mental healthcare training impacts stigmatizing attitudes and behaviors. Providers were trained using either a standard mhGAP approach (training-as-usual arm) or through a training involving mhGAP plus social contact with service users, caregivers, and aspirational figures (RESHAPE arm).

Providers in both training arms reported having lacked knowledge, experience, and confidence with mental health prior to training, and some feared or had negative encounters with mental health patients. After training, they learned that mental illness can affect anyone, is treatable, and should be approached with empathy. Increases in willingness and confidence to treat mental illness were reported, which were attributed to increased knowledge and skills, as well as experiences seeing their own patients improve.

**Fig. 2:**
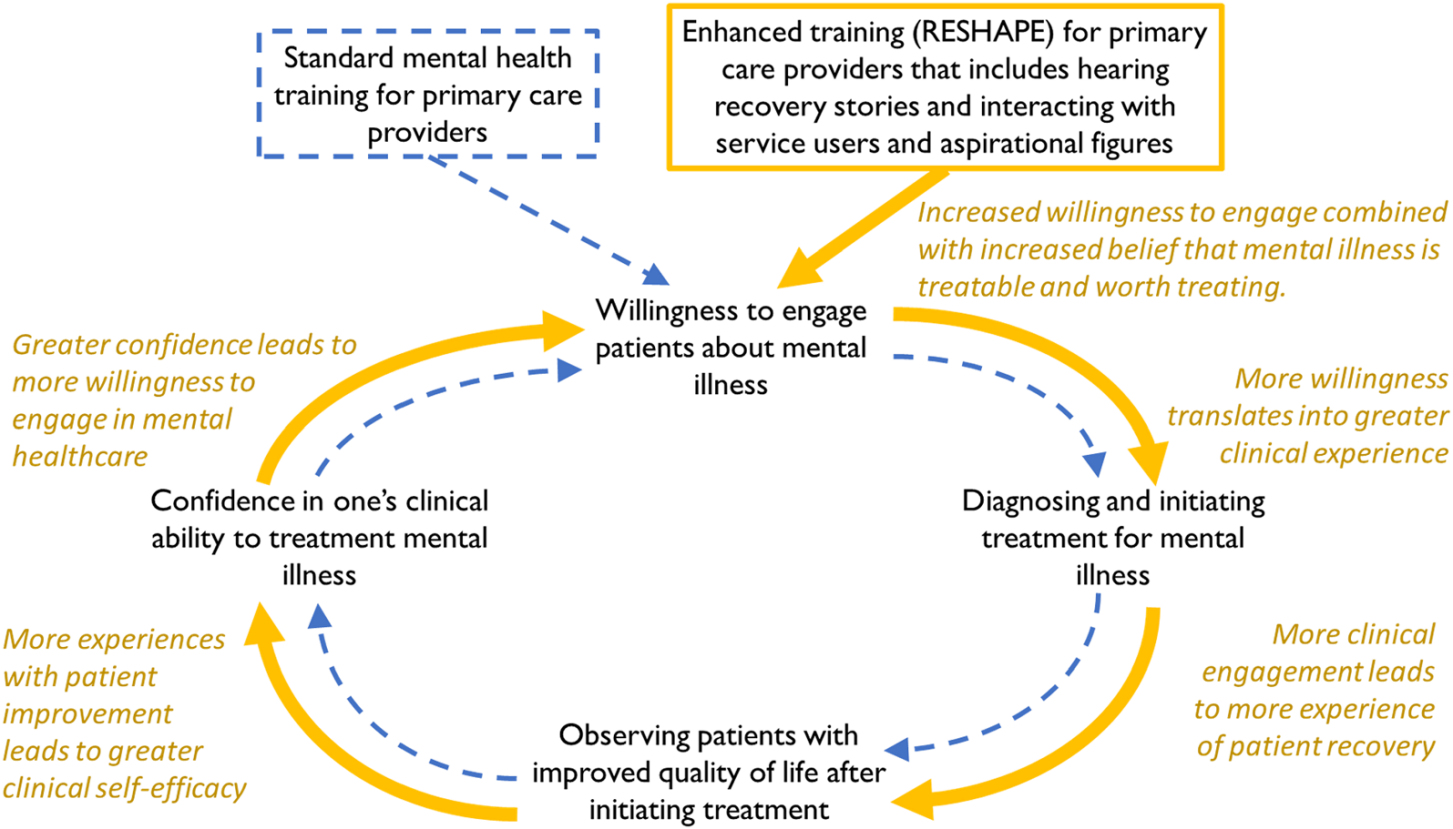
Positive feedback cycle created by early engagement with service users and aspirational figures during training

Although most qualitative themes overlapped between training arms, we identified some important differences between them. RESHAPE trainees were more likely to express willingness to treat mental health patients and to discuss concerns regarding patient interactions, such as ensuring patient safety and promoting openness. These changes align with previously reported quantitative outcomes, namely that RESHAPE trainees experienced a greater decrease in stigmatizing attitudes, saw more mental health patients, and were more accurate in diagnosing patients compared to TAU trainees [33]. RESHAPE trainees explicitly pointed to the involvement of service users and aspirational figures during training as an important aspect of their changed attitudes. Trainees in both arms described a positive feedback cycle of increased patient interaction, improved patient outcomes, and increased confidence and willingness to treat. This cycle appeared to be amplified for RESHAPE providers, contributing to ongoing stigma reduction and improvement of clinical skills (Fig. 2).

### Processes of change

#### From fear to empathy and recovery

Our findings are strongly aligned with previous studies regarding how stigma is manifested among primary care providers, as well as what works to reduce it. First, peril stigma and empathy featured prominently in interviews [19, 36, 61]. Providers emphasized experiences of fear and witnessed or anticipated violence prior to the training. They also described enacting increased social distance (e.g., refusing to see mental health patients, only referring them). Providers then described feeling more comfortable rather than fearful following the training.

Participants’ descriptions of changes following the training align with Knaak et al.’s [30] “key ingredients” for stigma-reduction trainings. Providers described experiencing greater empathy post-training, emphasizing that mental health patients should be treated with care and respect. Participants’ language in describing the changes that they experienced reflect a shift from a pathology-first perspective (e.g., referring to patients as “mad,” “psycho”) to a person-centered approach (e.g., saying they are people like us, calling them brothers and sisters). In contrast to providers’ descriptions of violence and negative encounters pre-training, service users who spoke during the RESHAPE training were often described as being “like us.” These findings agree with existing literature regarding the significance of reducing anxiety and increasing empathy for effective stigma-reduction activities [19, 48].

Another “key ingredient” [30] that featured in interviews was an emphasis on recovery, as most trainees noted learning that mental health patients can be treated and return to a “normal” life. Many interviewees contrasted this with their perception prior to the training, when they thought that mental illness was permanent and untreatable. Knowing that those service users were treated not by an expert psychiatrist but someone with similar training like them also might have increased their belief, confidence, and willingness to treat mental illness.

#### Knowledge and skills build confidence

Participants emphasized that changes in attitudes and behavior were largely attributable to having gained knowledge and skills for treating mental illness. Shortcomings in knowledge, attitudes, and confidence have been identified as important areas that each must be addressed to improve mental health treatment-provision by primary care providers [17, 38]. Participants in our study described having believed several forms of misinformation that aligned with the myth-busting activity; although no participants referred to myth-busting explicitly, providers’ statements addressed most of the myths discussed during the training. For example, the notion that mental illness can be treated was one of the most common things that participants said they learned from the training.

Before being trained, most providers felt ill-equipped to provide mental healthcare, noting that they did not know how to talk to patients, let alone treat them. After the training, they described knowing how to speak with patients in a genuine caring and non-stigmatizing manner, how to provide counseling, and how to provide pharmaceutical treatment.

Participants emphasized confidence developed through the training, which they attributed to knowledge and skills gained, as well as positive experiences seeing patients improve. Specific statements regarding skills learned were more common among RESHAPE participants. Quantitative findings from the overall study likewise found that RESHAPE trainees experienced greater increases in self-efficacy [33]. These findings add to the literature regarding the importance of knowledge and self-efficacy for stigma reduction among healthcare providers [2, 19, 22, 30, 48].

#### Willingness to treat

Interview participants expressed willingness to treat mental health patients, which is consistent with provider self-reports in diverse global settings [36]. However, in other settings, the willingness appears not to translate into action because of other beliefs that mental illness is not treatable, that mental health patients and their families are too unpredictable to follow-through on care, and the fear of violence that prevents acting on the willingness. Yet, the RESHAPE providers in this study strongly endorsed a willingness and a change in attitudes, such as treatability of mental illness, which together led to actually engaging in care-provision. Although interview data might only reflect intended behavior, participants often described particular individuals—such as neighbors or patients—whom they had treated since the training. Additionally, these differences were sustained in behavioral outcomes. RESHAPE providers treated 38% more patients in the 1.5–2 years following the training and produced fewer false-positive diagnoses: 66% false positives in TAU, 28% false positives in RESHAPE [33].

Providers’ lack of willingness to treat mental illness has been identified as a key barrier to success of task-shifting interventions. For example, Ola and colleagues [44] assessed attitudes of Nigerian primary care providers attending a workshop on recognizing and treating common mental disorders. Although just over half of providers reported feeling comfortable caring for depressed patients, they also reported such care provision as not rewarding, that primary care workers could not usefully treat depression, and that psychotherapy should be left to specialists. Additionally, in Tunisia—where mental healthcare has been part of primary care since the 1990s—most primary care providers reported not feeling confident treating mental health patients, particularly those with psychosis, substance use, or suicide-related behaviors [54]. Considering primary care providers are the target group most often expected to deliver mental healthcare through task-shifting interventions, identifying ways to improve confidence and willingness to treat is key.

It remains unclear why willingness to treat represents the most marked difference between training arms, since upstream factors (namely knowledge and attitudes) did not differ as significantly between the groups in our qualitative data. This might represent social desirability bias, with providers in both training arms presenting themselves as having non-stigmatizing attitudes because they knew from the training how they were expected to feel towards patients. Indeed, the pilot trial found that attitudes did differ between training arms as assessed using the Social Distance Scale [33], which is completed individually and therefore less prone to social desirability bias.

Service user testimonials in the RESHAPE arm likely strengthened the belief that mental health patients can improve, particularly because those service users came from the same community providers worked in. RESHAPE participants’ descriptions of what they found most valuable from the training reflect the key role of service users. Specifically, participants emphasized the positive impact of hearing personal testimony, both from the service users and caregivers, as well as providers who cared for them.

These findings suggest that the key difference between TAU and RESHAPE—social contact with service users and aspirational figures—might play an important role in translating changes in knowledge, attitudes, and confidence into willingness to treat. Willingness to treat might be an important mechanism of action to translate changes in knowledge and attitudes into changes in behavior, which have proven far more difficult to achieve in stigma-reduction interventions [14, 12, 20, 22, 56].

### Limitations and future directions

Stigma was explicitly discussed during the training, meaning that participants likely knew what the “right answers” were during interviews. Additionally, one of the interviewers was involved in some of the training sessions, with a focus on research aspects. It might be the case that responses—particularly in the interviews—reflect social desirability, which might account for the limited differences seen in qualitative descriptions despite quantitative differences found between training arms and in SDS scores.

Differences in sample sizes between the training arms might have resulted in a greater likelihood of themes arising in interviews with the RESHAPE providers compared to TAU providers. However, other than mentioning social contact aspects of the training, there were no themes that were raised only by RESHAPE and not TAU providers. We were also careful not to focus our subgroup comparisons on counts but on proportionality of themes between the two training arms.

Our findings reflect a strong sense among trainees that anyone can get mental illness. Interestingly, for some participants, this knowledge that anyone can become mentally ill seemed to extend to a concern that they themselves could become mentally ill after treating patients, suggesting that some myths might persist even after training. Providers described having raised such concerns during the training and receiving reassurances that they could be treated, as well as specific self-care skills to help them avoid experiencing mental illness. Additionally, although most providers reported not having experienced stigma themselves, some participants seemed to suggest they might anticipate experiencing it in future. Attending to providers’ own experiences of stigma is an important component of such trainings [19]. This study focused on interpersonal stigma, but it is also important to address structural stigma [22], which can potentially be obscured by interventions focusing only on interpersonal aspects of stigma [20].

Future trainings should attend to these concerns and perhaps provide additional time addressing this myth. There are currently studies underway to test the RESHAPE strategy in other settings [59]. In addition, a full-scale trial is currently being conducted in Nepal [32].

Based on these preliminary findings, it is worthwhile to consider what could be done with current mhGAP and other mental health trainings in primary care. Our qualitative findings about mechanisms of change support existing quantitative research suggesting:

1. People with lived experience of mental illness should be considered as part of the training team alongside mental health specialists.
2. Primary care providers who have successfully taken on mental health services should be involved as role models for PCPs in training.
3. In addition to the knowledge focus of mhGAP, attention should be paid toward drawing upon lived experience of service users and aspirational figures to demonstrate messages of treatability and recovery from mental illness.

## Conclusions

There are increasing efforts to reduce stigma among primary care providers, an important target of mental health task-shifting interventions globally [21]. Two of the most significant challenges are altering providers’ willingness to provide care [44] and altering behavior [14, 56]. We found that primary care providers who receive mental health training are likely to report improvements in knowledge and skills but will not necessarily experience changes in willingness to provide treatment or desire for less social distance from mental health patients. The pathway emerging from our study is that stigma reduction—which was achieved through building knowledge and skills and facilitating social contact to demonstrate recovery and promote empathy—leads to greater willingness to engage with people with mental illness, which leads to more positive experiences of patients who benefit from mental healthcare. Training approaches that incorporate social contact with service users in recovery might be key to improving beliefs, such as mental illness is treatable and worth treating, and translating them into increased willingness to treat and ultimately into increased care provision. Therefore, whereas mhGAP may provide the key knowledge and skills, initiatives such as collaboration with service users are needed to effectively translate mhGAP clinical knowledge into actions that can transform the quality of life of their patients. Future research should focus on behavior change, including not only whether providers are increasingly delivering mental healthcare but whether the care is high quality. Service user perspectives will be central to this research and implementation.

## Supplementary Information

**Additional file 1**. Interview guide in English and Nepali.

## Data Availability

The datasets used and/or analysed during the current study are available from the corresponding author on reasonable request.

## Abbreviations

HICs: High-Income Countries
LMICs: Low- and Middle-Income Countries
mhGAP: mental health Gap Action Programme
PCP: Primary Care Provider
PRIME: Programme to Improve MEntal Healthcare
RCT: Randomized Control Trial
RESHAPE: REducing Stigma among HealthcAre Providers
SDS: Social Distance Scale
TAU: Training-As-Usual

## Footnotes

1 Refers to change in Social Distance Scale (SDS) score from pre- to post-training.

2 “Care” is most often discussed in a way that relates it to emotionally sincere caring and support, as exemplified by the “care” a mother would show her child (*maya-mamata*).
