# Supplementary material for: Mechanisms of action for stigma reduction among primary care providers following social contact with service users and aspirational figures in Nepal: an explanatory qualitative design
Source: Int J Ment Health Syst. 2022 Aug 11;16:37. doi: 10.1186/s13033-022-00546-7 (PMC9367153; doi:10.1186/s13033-022-00546-7)
Supplement: Supplementary file 1 — Additional file 1. Interview guide in English and Nepali. [file 13033_2022_546_MOESM1_ESM.pdf]

Primary Care Trainees [Prescribers & Non-prescribers] (direct beneficiaries) प्राथमिक सेवा प्रशिक्षार्थी[Prescribers & Non-prescribers] (प्रत्यक्ष लाभान्वितहरु) – Qualitative Interview Guide

English	Nepali
1. What was your overall experience of the training?	१. यस तालिममा तपाईको समग्र अनुभव कस्तो रह्यो ? के के नयाँ कुराहरु सिक्नुभयो, आफनो कामलाई सहयोग पुरयाँउछ जस्तो लाग्यो?
2. How will this training impact how you think about or treat PWMI?	२. यस तालिमले मनको समस्या भएको विरामीहरु प्रति र उपचार गर्ने पद्धतिमा तपाईको विचारलाई कसरी प्रभाव गर्छ?
3. What could be improved about the training?	३. यस तालिममा के कस्ता सुधारहरु गर्न सकिन्छ ?
4. What are barriers to implementing mental healthcare in your facility?	४. तपाईको स्वास्थ्य केन्द्मा मनोसामाजि सेवा प्रदान गर्न के कस्ता बाधाहरु छन् ?
5. What elements of mental healthcare do you feel comfortable doing?	५. मानसिक स्वास्थ्य सेवा सम्बन्धि तपाईलाई कुन कुन कुराहरु गर्न सहज महसुस भयो ?
6. What areas would you like further training in?	६. कुन कुन विषयमा तपाईलाई थप तालिमको चाहाना छ ?
7. What are the potential reasons to provide mental health services at your health facility?	७. तपाईको स्वास्थ्य चौकीमा मानसिक स्वास्थ्य सेवा प्रदान गर्नु पर्ने कारणहरु के के होला ?
8. What are potential reasons to not provide mental health services at your facility?	८. तपाईको स्वास्थ्य चौकीमा मानसिक स्वास्थ्य सेवा प्रदान गर्नु नपर्ने कारणहरु के के होला ?

9. What other work is negatively impacted by providing mental health services?	९. मानसिक स्वास्थ्य सेवा प्रदान गर्दा अन्य कुनकुन कामहरुमा नकरात्मक असर पर्छ?
10. Are there any personal benefits or negative consequences to you and your family through participation in mental health services? Is so, please give examples.	१०. मानसिक स्वास्थ्य सेवा प्रदान गर्दा तपाई तथा तपाईको परिवारलाई कुनै व्यक्तिगत लाभ अथवा हानी हुन्छ? यदी हुन्छ भने उदाहरण दिन सक्नुहुन्छ?
11. What was your experience of completing the pre- and post-tests?<br>a. What was your experience of the written test?<br>b. What was your experience of the role play (ENACT)?<br>c. What was your experience of the computer game?<br>d. What was the reason that you and other trainees were given these tests?	११. तालिम पूर्व तथा तालिम पश्चातको जाँचमा तपाईको सहभागीता बारे तपाईको अनुभव कस्तो रह्यो ?<br>११.१ लिखित जाँच सम्बन्धित तपाईको अनुभव कस्तो थियो?<br>११.२ भुमिका प्रदर्शन भ्ल्ब्त्रत सम्बन्धित तपाईको अनुभव कस्तो थियो?<br>११.३ कम्पयुटर गेम सम्बन्धित तपाईको अनुभव कस्तो थियो?<br>११.४ तपाई तथा तालिमका अन्य सहभागीहरुलाई किन यि जाँच लिईएको थियो भनि तपाईले बुझ्नुभयो?
12. What have you learned from health workers at your facility or other facilities who took part in the trainings?	१२. यस तालिममा सहभागी भएका स्वास्थ्यकर्मी (तपाईले काम गर्ने स्वास्थ्यचौकी अथवा अरु स्वास्थ्यचौकी) हरुबाट तपाईले के सिक्नुभयो?
13. [If RESHAPE-mh arm] What was your experience of PWMI participating in the training?<br>a. What did PWMI do at the training?<br>b. What were the benefits of having PWMI take part in the training?	१३[If RESHAPE arm] यस तालिममा मनको समस्या भएको व्यक्तिहरु सहभागी हुँदा तपाईको अनुभव कस्तो रह्यो?<br>१३.१ उहाँहरुले तालिममा के गर्नुभयो?<br>१३.२ उहाँहरु सहभागी हुदाँका फाइदाहरु के के थिए?<br>१३.३ उहाँहरु सहभागी हुदाँका बेफाईदाहरु के के थिए?

c. What were the drawbacks of having PWMI take part in the training?<br>d. How could participation of PWMI at the trainings be improved?<br>e. How could PWMI help in other areas of your training, supervision, or work?	१३.४ उहाँहरुले तालिम, सुपरिवेक्षण तथा अन्य कार्यमा कसरी सहयोग गर्न सक्छन् होला?
